# Gelation Behavior of 5-Chloro-8-hydroxyquinoline, an Antituberculosis Agent in Aqueous Alcohol Solutions

**DOI:** 10.3390/antibiotics1010017

**Published:** 2012-09-19

**Authors:** Erkki Kolehmainen, Hannu Salo, Jukka Korpela

**Affiliations:** Department of Chemistry, University of Jyväskylä, P.O. Box 35, FIN-40014 Jyväskylä, Finland; Email: hannu.t.salo@jyu.fi (H.S.); jukka.p.korpela@jyu.fi (J.K.)

**Keywords:** gel, 5-chloro-8-hydroxyquinoline, *Mycobacterium tuberculosis*

## Abstract

It was shown that 5-chloro-8-hydroxyquinoline, an antituberculosis agent, gels aqueous alcohol solutions efficiently. Thermal stability and gel-to-sol transition temperature of 1% gel in CD_3_OD/D_2_O (2:1) was studied by ^1^H-NMR. Fibrous structures of four xerogels have been characterized by scanning electron microscope.

## 1. Introduction

Tuberculosis (TB) is one of the most serious infectious diseases caused by a single pathogen. One-third of the human population is reported to be latently infected with *Mycobacterium tuberculosis*, and millions of lives are lost every year [1].

In addition to many known antituberculosis drugs in use [2], 5-chloro-8-hydroxyquinoline or 5-chloroquinol-8-ol (Cloxyquine) has shown to incur *in vitro* activities against *Mycobacterium tuberculosis* [3]. Also, 8-hydroxyquinoline itself is effective against *Mycobacterium tuberculosis *[4] and some derivatives are shown to act as antibacterial agents that target intra- and extracellular Gram-negative pathogens [5]. Further, a novel polymorph of 5-chloro-8-hydroxyquinoline with improved water solubility and faster dissolution rate has been reported which can improve the bioavailability of the drug [6]. In addition, tacrine-8-hydroxyquinoline hybrids have been shown to have potential as multifunctional agents for the treatment of Alzheimer’s disease and have copper(II) complexing properties [7]. It has also been recently shown that some related mixed ligand transition metal complexes have antituberculosis activity [8]. The importance of mixed ligand transition metal complexes in drug development is further, more generally, described [9,10] 

Drug resistance in *Mycobacterium tuberculosis* was shown to be related to pharmacokinetic/pharmacodynamic (PK/PD) factors [11]. Therefore the gelation ability of this potential drug in aqueous solution can be of extreme importance owing to its usability in controlled release applications [12]. 

In conjunction with our interest in low molecular weight supramolecular (self assembling) gelators [13] we are now reporting our study on the gelation behavior of 5-chloro-8-hydroxyquinoline in aqueous alcohol solutions. These gelation properties can probably be related also with the supramolecular synthon pattern in solid cloxiquine, reported recently [14]. We consider that these results can help in tailoring better drug delivery and pharmaceutical formulation combating tuberculosis. 

## 2. Results and Discussion

**Figure 1:**
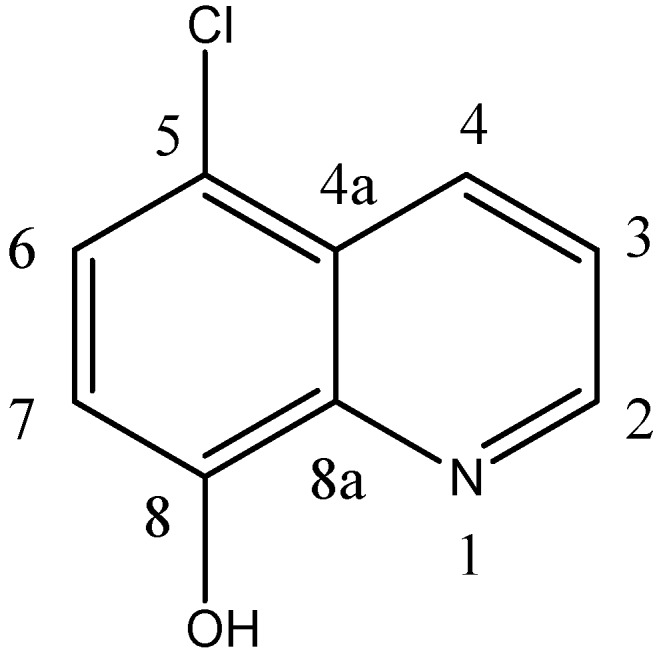
The structure and numbering of 5-chloro-8-hydroxyquinoline.

Figure 1 shows the structure and numbering of 5-chloro-8-hydroxyquinoline.

Table 1 lists the gelation data in various aqueous alcohol solutions at 295 K. The gel formation was observed by the test tube inversion method. Systematic temperature studies of all gels were not performed. However, for 1% 5-chloro-8-hydroxyquinoline gel in CD_3_OD/D_2_O (2:1) the gel-to-sol temperature was between +28 °C and +30 °C observed by ^1^H-NMR.

**Table 1:** Gelation data of 5-chloro-8-hydroxyquinoline (cloxiquine).

Entry	Solvent system	[cloxiquine] gel stability
1	1:1 EtOH/H_2_O	1.0% very stable
2	1:1 EtOH/H_2_O	0.75% stable
3	1:1 EtOH/H_2_O	0.5% stable
4	1:2 EtOH/H_2_O	1.0% stable
5	1:2 EtOH/H_2_O	0.5% stable
6	1:2 EtOH/H_2_O	0.2% poor
7	1:4 EtOH/H_2_O	1.0% stable
8	2:1 MeOH/H_2_O	1.0% very stable
9	2:1 MeOH/H_2_O	0.75% stable
10	2:1 MeOH/H_2_O	0.5% precipitate
11	1:2 MeOH/H_2_O	1.0% precipitate
12	1:4 MeOH/H_2_O	1.0% precipitate
13	2:1 1-PrOH/H_2_O	1.0% precipitate
14	1:1 1-PrOH/H_2_O	2.0% very stable
15	1:1 1-PrOH/H_2_O	1.0% stable
16	1:1 1-PrOH/H_2_O	0.75% poor
17	1:1 1-PrOH/H_2_O	0.5% precipitate
18	1:2 1-PrOH/H_2_O	1.0% stable
19	1:4 1-PrOH/H_2_O	1.0% stable
20	1:4 1-PrOH/H_2_O	0,5% stable
21	1:4 1-PrOH/H_2_O	0.2% stable
22	1:4 1-PrOH/H_2_O	0.1% stable
23	1:8 1-PrOH/H_2_O	1.0% precipitate
24	1:10 1-PrOH/H_2_O	1.0% precipitate
25	1:1 2-PrOH/H_2_O	2.0% stable
26	1:1 2-PrOH/H_2_O	1.0% stable
27	1:1 2-PrOH/H_2_O	0.5% stable
28	1:1 2-PrOH/H_2_O	0.2% poor
29	1:2 2-PrOH/H_2_O	1% (g) stable
30	1:4 2-PrOH/H_2_O	1% precipitate

As can be seen, increasing the concentration of 5-chloro-8-hydroxyquinoline results in a better stability of the formed gels. However, 5-chloro-8-hydroxyquinoline is not a real hydrogelator because increasing the water molar ratio generally results in a precipitate formation.

**Figure 2:**
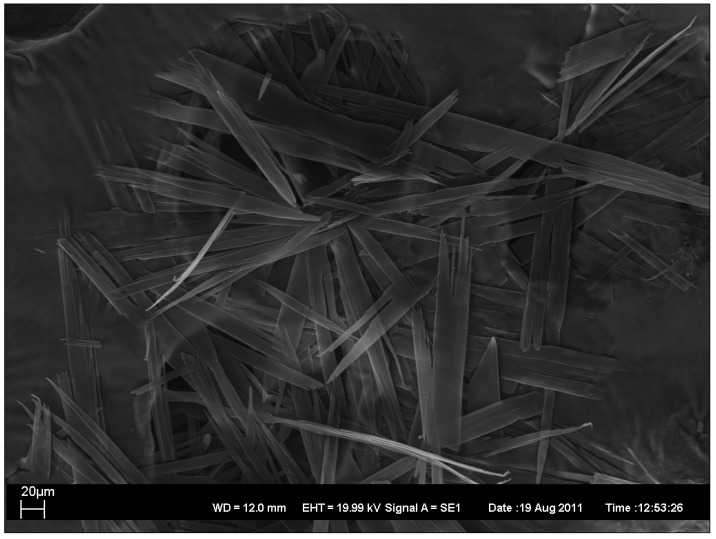
SEM image of xerogel from 1:1 EtOH/water 1% gel.

**Figure 3:**
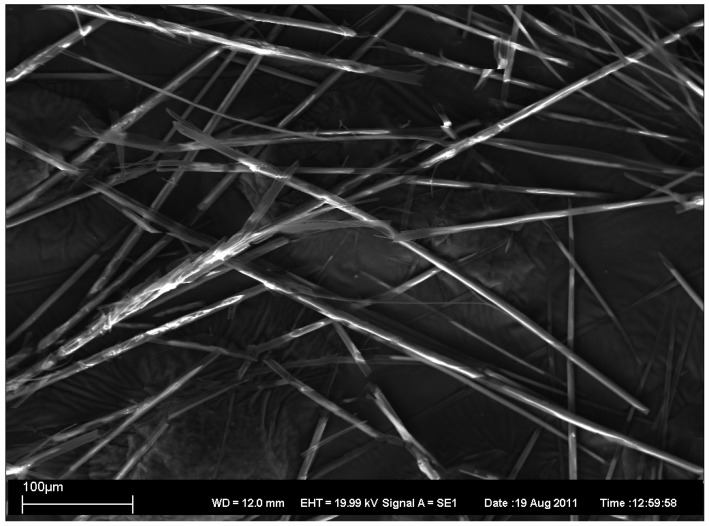
SEM image of xerogel from 2:1 MeOH/water 1% gel.

**Figure 4:**
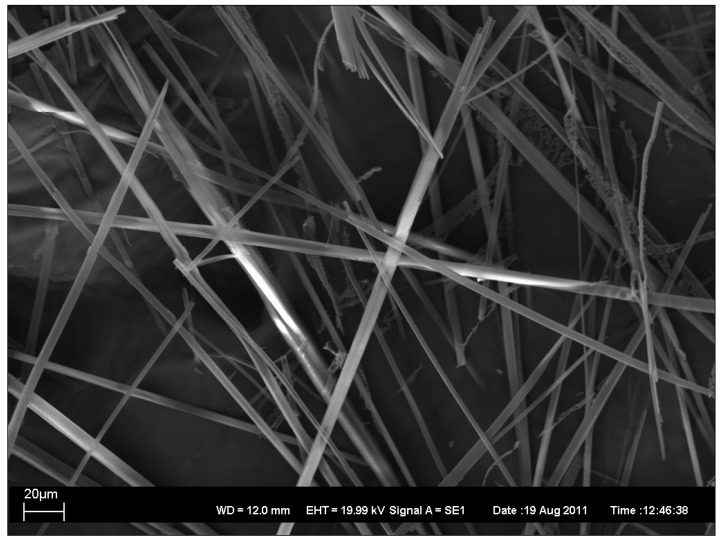
SEM image of xerogel from 1:1 1-PrOH/water 2% gel.

**Figure 5:**
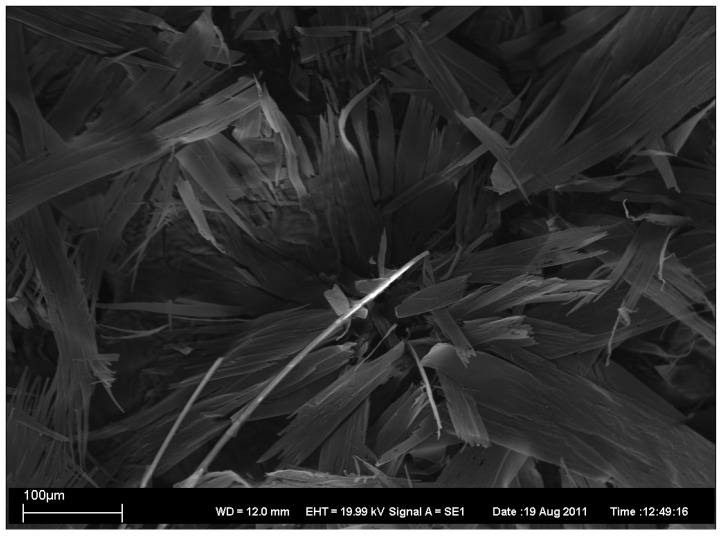
SEM image of xerogel from 1:1 1-PrOH/water 1% gel.

In addition, the topography of the gels was characterized by the electron microscopy. Four xerogels have been characterized by scanning electron microscopy (SEM), as described in Figure 2, Figure 3, Figure 4, Figure 5. 

As can be seen, SEM images 2–5 revealed the presence of long rod-like structures with variable rod diameters. However, there exists some variation in the width and the length of the rods. In xerogels from 1:1 EtOH/water 1% gel (Figure 2) and from 1:1 1-PrOH/water 1% gel (Figure 5) the width variation is clearly larger and the average widths is greater than in the others (Figure 3 and Figure 4) which also show less bundled structures. In all xerogels, rods reveal no or very weak branching and the overall structure can thus be considered as an open-type network. The fractures of the network seen in Figure 5 are probably due to the faster evaporation of the solvent.

**Figure 6:**
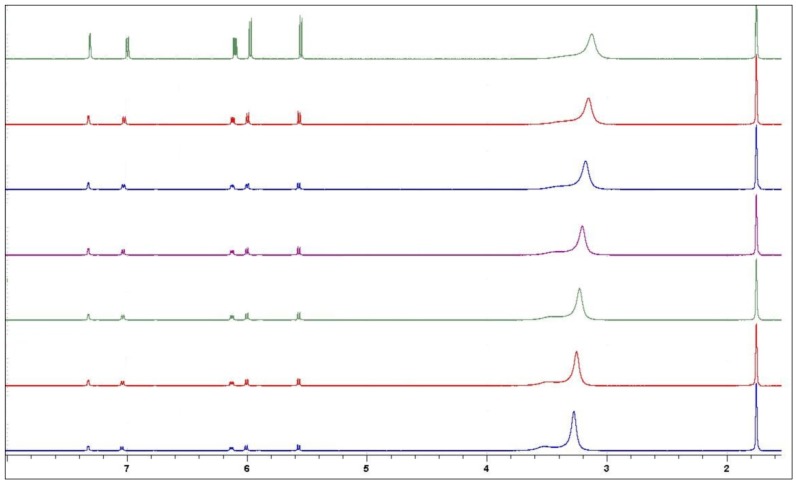
Variable temperature 500 MHz ^1^H-NMR spectra of 1.0% 5-chloro-8-hydroxy-quinoline in CD_3_OD/D_2_O (2:1) from +18 °C (bottom) to +30 °C (top) in 2 °C steps. The clearincrease in the signal intensity between +28 °C and +30 °C is due to the gel-to-sol transformation.

The gel-to-sol transformation was studied by variable temperature NMR. This technique was successfully used recently in gel-to-sol transformation studies [15]. Figure 6 describes 500 MHz ^1^H-NMR spectra of 1.0% 5-chloro-8-hydroxyquinoline in CD_3_OD/D_2_O (2:1) from +18 °C (bottom) to +30 °C (top) in 2 °C steps. As can be seen, a clear increase in the signal intensity happens between +28 °C and +30 °C. This means that the number of motionally limited or NMR “silent” molecules of the gel [16] decreases significantly between these two temperatures owing to the gel-to-sol transformation where the molecular motion of 5-chloro-8-hydroxyquinoline is no longer restricted. Although this gel-to-sol change happens below the physiological conditions this finding suggests that the gel stability could be improved by other modifications of solvent systems. 

## 3. Experimental

5-Chloro-8-hydroxyquinoline (95%) was purchased from Sigma-Aldrich and used without purification because its ^1^H-NMR spectrum did not reveal any impurity signals [17]. All solvents were also from commercial sources and used as obtained. Deionized water was from our own laboratory. The samples were prepared by weighing an accurate amount of 5-chloro-8-hydroxyquinoline in a known volume of the solvent system in a test tube. After that the mixture was heated in a water bath until the solute dissolves. Then the test tube was closed and allowed to cool to room temperature. The gel formation was detected by the tube inversion technique as non-mobility of the sample and by visual inspection.

SEM images have been taken with Zeiss EVO 50 scanning electron microscope. In Figure 2, Figure 3, Figure 4, Figure 5 are also given the working distance 12.0 mm and acceleration voltage 19.99 kV.

VT ^1^H-NMR spectra were run with Bruker Avance DRX 500 FT NMR spectrometer in CD_3_OD/D_2_O 2:1-mixture using 30° flip angle and 4 scans. The chemical shift scale is referenced to the trace signal of CHD_2_OD at 3.31 ppm from internal TMS.

## 4. Conclusions

It was shown that 5-chloro-8-hydroxyquinoline, an antituberculosis agent, efficiently gels aqueous alcohol solutions. A variable temperature ^1^H-NMR study reveals that the gel-to-sol transition of 1.0% 5-chloro-8-hydroxy-12-quinoline in CD_3_OD/D_2_O (2:1) happens between +28 °C and +30 °C. Although this gel-to-sol transition takes place below the physiological conditions, this finding suggests that the gel stability could be improved by other modifications of solvent systems. This finding can be useful for the drug delivery and preparation of pharmaceutical formulations of 5-chloro-8-hydroxyquinoline.
